# Acceptability of a Behavioral Activation Intervention for Functioning in Older Veterans

**DOI:** 10.1093/geroni/igaf122.2817

**Published:** 2025-12-31

**Authors:** Meaghan Kennedy, Sydney Ruggles, Christina Cardwell, Anastasia Canell, Jaye McLaren, Kathleen Lyons, Jonathan Bean, Megan Kelly

**Affiliations:** VA Bedford Health Care System, Bedford, Massachusetts, United States; VA Bedford Health Care System, Bedford, Massachusetts, United States; Lemuel Shattuck Hospital, Boston, Massachusetts, United States; VA Bedford Health Care System, Bedford, Massachusetts, United States; VA Bedford Healthcare System, Bedford, Massachusetts, United States; Massachusetts General Hospital Institute of Health Professions, Boston, Massachusetts, United States; Harvard Medical School, Boston, Massachusetts, United States; University of Massachusetts Chan Medical School, Worcester, Massachusetts, United States

## Abstract

Behavioral activation (BA) is an evidence-based psychotherapy for depression that also shows promise as a transdiagnostic intervention to support functioning. We adapted a brief BA protocol to improve physical, cognitive, and social functioning in older Veterans at risk for decline. In this study, we conducted an open pilot to assess acceptability of the adapted intervention among 10 older (age ≥65) Veterans at risk for functional decline (Vulnerable Elders Survey-13 score ≥3). Participants completed a 6-session telehealth-delivered BA intervention, baseline and post-intervention assessments, and a semi-structured interview based on the Theoretical Framework of Acceptability. Interviews were analyzed by two researchers using rapid qualitative analysis. Participants had a mean age of 79.6 years (range 69-88) and 80% were male. Nine participants completed all 6 intervention sessions and follow-up assessments; one withdrew after the second session. Mean satisfaction on the Client Satisfaction Questionnaire (CSQ-8) was 26.9±3.7 (on a scale of 8-32). Participants found the telehealth format convenient and the length and frequency appropriate. The content was highly aligned with participants’ values and what mattered most to them. While most participants found value in tracking their activities, others found it tedious and recommended simplifying the process. The intervention helped participants stay organized, set goals, and break tasks into parts; increased their motivation and helped them refocus on valued activities; and provided them with skills they will continue to use to support activity engagement. Our study suggests that a brief telehealth-delivered BA intervention for functioning is acceptable, values-aligned, and perceived as effective among older Veterans.

